# Safeguarding youth from agricultural injury and illness: Perspectives from Burkina Faso

**DOI:** 10.3389/fpubh.2022.984988

**Published:** 2022-08-29

**Authors:** Windkouni H. Eugenie Maïga, Salimata Traoré

**Affiliations:** ^1^Unité de Formation et de Recherche en Sciences Economiques et de Gestion (UFR-SEG), Université Norbert Zongo, Koudougou, Burkina Faso; ^2^Unité de Formation et de Recherche en Sciences Economiques et de Gestion (UFR-SEG), Université Thomas SANKARA, Saaba, Burkina Faso

**Keywords:** youth, agriculture, safety, health, Burkina Faso

## Current situation

In developing countries, the agricultural sector is the main provider of employment for the majority of the population (1). In Burkina Faso, this sector occupies 75% of the working population who live mostly in rural areas (2). Burkina Faso is a landlocked country located in the heart of West Africa with an estimated population of about 20 and a half million (3), according to the 2019 population census. The major types of agriculture are crop production, animal husbandry, and exploitation of non-timber forest products (NTFPs). Crop production is dominated by sorghum, millet, corn and rice, the main food crops, and cotton, which is the main cash crop. Most farms are family-based, small, and located in rural areas, utilizing numerous child workers daily. These farmers practice subsistence agriculture in a context where the rural area contributes the most to poverty in the country at 91.8% (2).

According to data from the 2006 National Child Labor Survey (4), 41.1% of children aged 5 to 17 are economically active in Burkina Faso. Of these, 95.8% are in child labor, with the agricultural sector being one of the most affected sectors at 69.2%. Regardless of the type of agricultural work, it involves several tasks that expose children to numerous risks. These risks are related to climatic conditions; the seasonal and physical nature of the activity; contact with animals and plants (bites, poisoning, infections, parasites, allergies, and intoxications); to exposure to chemical products. Unsafe pesticide use is a major problem in the agricultural sector in Burkina Faso (5), leading to illness or death. In September 2019, 18 people (13 from the same family) died from pesticide-related illness,^1^ while in 2016, a study revealed that 341 in three regions of Burkina Faso suffered from pesticide-related illnesses.^2^ The legal framework for pesticide use exists but the law is not enforced.

When children have a minimum legal age to work, the agricultural sector is the one that offers, beyond the daily risks, the most possibilities to work decently. Given the importance of the agricultural sector for the country's economic development, knowing the conditions for creating a safe environment for children is necessary for the implementation of good measures for their protection.

Burkina Faso recognizes the rights of children as the ratification of several conventions indicate. These include ILO Convention No. 5 on the Minimum Age for Admission to Employment in Industry, Convention No. 138 on the Minimum Age for Admission to Employment, and Convention No. 182 on the Worst Forms of Child Labor. No specific law regarding the health, safety, and wellbeing of children and adolescents on farms exists so far, but the country ratified the Convention No. 184 on health and safety in agriculture in 2009.

## Current activities

The national laws are in place to specifically protect children, including Law 11/92 of 22/12/1992 on the Labor Code, Order N°539 ITLS-HV of 29/07/1954 on child labor, and Order N°958 FPT/DGTLS of 07/10/1976 on the apprenticeship contract (6). Within the Ministry in charge of social affairs, the General Directorate for the Protection of Children and Adolescents was created to better ensure that children's rights are respected. This directorate is made up of three departments: the department for placements and adoptions; the department for the supervision and promotion of early childhood; and the department for the protection and fight against violence against children.

The Permanent Secretariat of the National Plan of Action for Children (SP/CNSPDE) has been set up to coordinate and monitor all actions to ensure that children's rights are respected. The secretariate has a website^3^ but it is not updated. A series of training courses and conferences on children's rights are held each year for staff working with and/or for children. These include social workers, health workers, magistrates, primary and secondary school teachers, police officers, gendarmes, prison security guards, students in vocational schools, customary and religious leaders, political and administrative authorities, leaders of associations, and NGOs. Since the start of the 2006–2007 academic year, a module on the rights of the child has been taught in national professional training schools for workers from justice, police, and social departments: Ecole Nationale d'Administration et de la Magistrature (ENAM), Ecole Nationale de la Police (ENP), Ecole Nationale de la Gendarmerie (ENG), and Institut National de Formation en Travail Social (INFTS).

In terms of activities, several initiatives are being taken in Burkina Faso. For example, a new national survey on child labor is underway to update the 2006 statistics. This is part of the implementation of the 2019–2023 National Strategy to combat the worst forms of child labor. In addition, Burkina Faso's national development framework, the National Economic and Social Development Plan, includes the fight against child labor as a priority; the objective was to "reduce the prevalence of children aged 5–17 involved in economic activities from 41% in 2006 to 25% in 2020.” An evaluation is underway to assess the achievements.

Non-governmental organizations have also taken initiatives to help protect children on farms. For instance, Terre des Hommes has supported the creation and implementation of community watch bodies named “Comités villageois de surveillance, CSV” (village surveillance committees) in two communes of Kénédougou province renowned for their orchard production to contribute to government efforts to curb child worst form of labor (7). The CSVs work on raising awareness about child protection from the worst forms of labor and developed community outreach, as well as mechanisms for controlling the movement of children.

## Current challenges

Improving the health, safety, and wellbeing of children and adolescents on farms in Burkina Faso comes with enormous and multi-faceted challenges. We identify five main categories of factors, namely, economic stability, education, social and community context, health and health care, and the neighborhood and built environment.

Poverty incidence in Burkina Faso remains high (41.4% in 2018) with the rural sector as the largest contributor (91.8%) (2). Poor households tend to use family labor much more than non-poor households; the latter have more resources for labor hire. Under these conditions, the presence of child laborers on family farms contributing to agricultural labor is more common. The fight against poverty remains a major challenge in this case to ensure the safety and wellbeing of children in these households.

In a country, economic instability can create uncertainty and reduce investment opportunities, which are obstacles to growth and economic development. Burkina Faso has been living under a regime of exception since the beginning of 2022, following a political coup d'état that has led to certain sanctions, such as the suspension of the country from many regional and international organizations. The country faces a crisis marked by strong fluctuations in economic activity and inflation. The challenge is to reduce, as much as possible, the instability in the country while maintaining the capacity of the economy to improve living standards.

Another reason why many children are available for work on farms is because of low school enrollment rates. Indeed, in the area of education, the challenges are enormous, as the population is predominantly illiterate (61.7%). For young people aged 15–24, the illiteracy rate is 13.4% in urban areas compared to 53.6.4% in rural areas (2). Policies to improve school enrollment rates should reduce the presence of children in the fields.

Beyond poverty, instability, and illiteracy, one of the biggest challenges is the social and community context. The Sahelian states have been experiencing unprecedented security and humanitarian crisis for several years, especially since the war in Libya. These countries are subject to recurrent attacks that make travel in some areas quite risky. Many people continue to flee and move to more secure areas. According to the United Nations High Commissioner for Refugees, there were more than 1.5 million internally displaced persons (IDPs) by the end of 2021, and Burkina Faso alone now accounts for 60% of the Sahel region's IDPs. As of the end of April 2022, there are 4,148 school closed because of insecurity, which means many children are forced to be out of school^4^. Therefore, there is a need to step up efforts to bring back stability for the proposed solutions to be sustainable.

## Future direction

Current data collection gives hope for future policy interventions regarding measures to improve the health, safety, and wellbeing of children involved in agricultural activities. To improve health, safety, and wellbeing of children, we must first document the prevalence of injuries that children sustain while being involved in agricultural activities. Several ministries should be involved in collecting this information on a regular basis: the ministry of health, the ministry of social affairs, the ministry of labor and youth, and the ministry of agriculture.

Documenting the issue should be followed by disseminating the information through websites, radio, TV, and live discussion in communities, where the prevalence of injuries is highest. Initiatives like the NGO Terre des Hommes' village surveillance committees should be encouraged in other parts of the country. Empowering local communities to develop endogenous mechanisms for protecting children involved in agricultural activities is one of the best ways to ensure sustainability of the actions taken. Training sessions for employers and farming families about the best practices on the use of pesticides, as well as safety measures to take on use agricultural equipment, would help reduce the number of injuries.

A specific law regarding health, safety, and wellbeing of children and adolescents on farms should be enacted to boost the existing general provisions on safety in agriculture. Yearly reports on the number of injuries sustained by children while working in agriculture-related activities should be produced and the trend should be monitored to take additional measures if needed.

Funding for supporting the different initiatives to protect child involved in agricultural activities should be sourced from taxes on imported pesticides. Ultimately, government vision and commitment to child safety in agriculture will be the key in securing the funding needed to implement the devised measures.

## Author contributions

WM and ST conducted the literature search. WM drafted sections Current activities and Future direction. ST drafted sections Current situation and Current challenges. All authors contributed to the full manuscript writing.

## Conflict of interest

The authors declare that the research was conducted in the absence of any commercial or financial relationships that could be construed as a potential conflict of interest.

## Publisher's note

All claims expressed in this article are solely those of the authors and do not necessarily represent those of their affiliated organizations, or those of the publisher, the editors and the reviewers. Any product that may be evaluated in this article, or claim that may be made by its manufacturer, is not guaranteed or endorsed by the publisher.

## Footnotes

^1^https://www.bbc.com/afrique/monde-49669368#:~:text=Un%20fermier%20africain%20r%C3%A9pand%20des,la%20ministre%20de%20la%20sant%C3%A9 (accessed June 28, 2022).

^2^https://cenozo.org/consommation-de-legumes-au-burkina-faso-ce-danger-lie-aux-pesticides-du-coton/ (accessed June 29, 2022).

^3^https://www.sp-cnspe.gov.bf/#:~:text=Le%20SP%2FCNSPDE%20est%20plac%C3%A9,l'Enfant%20(CNSPDE) (accessed June 25, 2022).

^4^https://www.panapress.com/4148-ecoles-fermees-a-cause-du-t-a_630721462-lang1.html (accessed June 29, 2022).
